# Mental health in children and young people with psoriasis. A comorbidity to consider

**DOI:** 10.1192/j.eurpsy.2025.1123

**Published:** 2025-08-26

**Authors:** M. Del Valle Martín, D. Velilla Antolin, N. Román Avezuela

**Affiliations:** 1 Psiquiatría, Hospital Universitario Severo Ochoa, Leganés; 2 Pediatría, Hospital Universitario Rey Juan Carlos, Móstoles; 3 Psiquiatría, Clínica San Miguel, Madrid, Spain

## Abstract

**Introduction:**

Children and adolescents with chronic cutaneous conditions are at risk of experiencing adverse psychosocial effects such as anxiety, depression, and loneliness. Children with psoriasis had significantly higher rates of any psychiatric disorder, but these are often unrecognised or under-recognised and not referred to mental health services. It is also clear that the well-being of these children’s families may also be impacted by their child’s condition.

**Objectives:**

The aim of this study was to review current knowledge of the comorbidity of psoriasis and psychiatric disorders in the paediatric population, which are often underdiagnosed and undertreated.

**Methods:**

A narrative literature review was carried out in the PubMed, Cochrane and Embase databases, selecting only the articles published in the last 10 years, using the following keywords: psoriasis, psychiatric disorders, paedriatic population.

**Results:**

There is no doubt that psoriasis is one of the most debilitating chronic dermatological conditions affecting children from a quality-of-life perspective. Indeed, numerous studies have demonstrated that its impact is on par with that of other chronic conditions such as diabetes, asthma or epilepsy. Current research generally supports a positive association between paediatric psoriasis and the onset of anxiety and depression. However, it is difficult to establish a causal relationship as there is some evidence that psoriasis and psychiatric illness can exacerbate each other. Children with psoriasis had significantly higher rates of any psychiatric condition, particularly depression and suicidal ideation. Patients with higher disease severity (Psoriasis Area and Severity Index (PASI) and Body Surface Area (BSA) scores) and longer disease duration, are undoubtedly more likely to experience worse anxiety and depression. They may have other psychiatric comorbidities, such as excoriation (skin picking) disorder or obsessive-compulsive disorder (OCD), resulting in body-focused repetitive behaviours that exacerbate psoriatic plaques.

Paediatric patients with psoriasis appear to be more vulnerable to the psychosocial effects of their disease than adults, especially those diagnosed at a younger age, who had poorer lifetime outcomes due to less robust coping skills compared to other children. Young children may also not be able to understand or express their emotions associated with psoriasis, which is a significant barrier to conducting research. There is little research on supportive treatment, but psychological support (group or individual) during appointments has been reported positively by patients.

**Conclusions:**

It is essential to consider the psychosocial impact of this particular pathology on children and their families, in order to improve their quality of life through a better understanding of their conditions and the implementation of interventions that help to mitigate these effects.

**Disclosure of Interest:**

None Declared

